# *PROS1* novel splice‐site variant decreases protein S expression in patients from two families with thrombotic disease

**DOI:** 10.1002/ccr3.1226

**Published:** 2017-11-03

**Authors:** Juliane Menezes, Célia Ventura, João Matos Costa, Elsa Parreira, Luísa Romão, João Gonçalves

**Affiliations:** ^1^ Department of Human Genetics Instituto Nacional de Saúde Doutor Ricardo Jorge Lisbon Portugal; ^2^ Gene Expression and Regulation Group Biosystems & Integrative Sciences Institute (BioISI) Faculdade de Ciências Universidade de Lisboa Lisbon Portugal; ^3^ Third Department of Internal Medicine Hospital Distrital de Santarém Santarém Portugal; ^4^ Hospital Fernando Fonseca Amadora Portugal; ^5^ Centre for Toxicogenomics and Human Health (ToxOmics) Genetics, Oncology and Human Toxicology Nova Medical School Universidade Nova de Lisboa Lisbon Portugal

**Keywords:** *PROS1*, protein S deficiency, thrombophilia, thrombosis, venous thromboembolism

## Abstract

Our results prove that c.1871‐14T>G is causative of type I PS deficiency, highlighting the importance of performing mRNA‐based studies in order to evaluate variants pathogenicity. We evidence the increased risk of venous thromboembolism associated with this cryptic splice‐site variant if present in patients with PS deficiency.

## Introduction

Protein S (PS) is a widely studied protein with an important function in the downregulation of thrombin formation. Since its discovery in 1976, more than 400 variants have been described in PS gene (*PROS1*) associated with PS deficiency and as a risk factor for venous thromboembolism (VTE). We describe a novel variant, c.1871‐14T>G, in intron 14 of *PROS1* gene identified in two patients with PS deficiency from two unrelated families with a history of thrombotic disease. This alteration leads to a *PROS1* mRNA expression reduction, probably due to nonsense‐mediated mRNA decay. Our results suggest that c.1871‐14T>G is causative of type I PS deficiency in these patients, highlighting the importance of screening not only the coding and the most conserved intron–exon junctions, but also perform mRNA‐based studies. We call attention to the potential increased risk of VTE in hereditary type I PS deficiency associated with this cryptic splice‐site variant.

## Report

Hereditary PS deficiency (PSD) is an autosomal dominant disorder associated with an increased risk of venous thromboembolism (VTE) 1, 2. The genetic basis of PSD is heterogeneous and is also associated with phenotypic variability. More than 400 variants in PROS1 have been described, the vast majority being missense or nonsense variants, distributed along the coding region 3. In this report, we describe a novel c.1871‐14T>G variant in *PROS1* intron 14, identified in two patients from two unrelated families, both with PSD and history of thrombotic disease.

**Figure 1:**
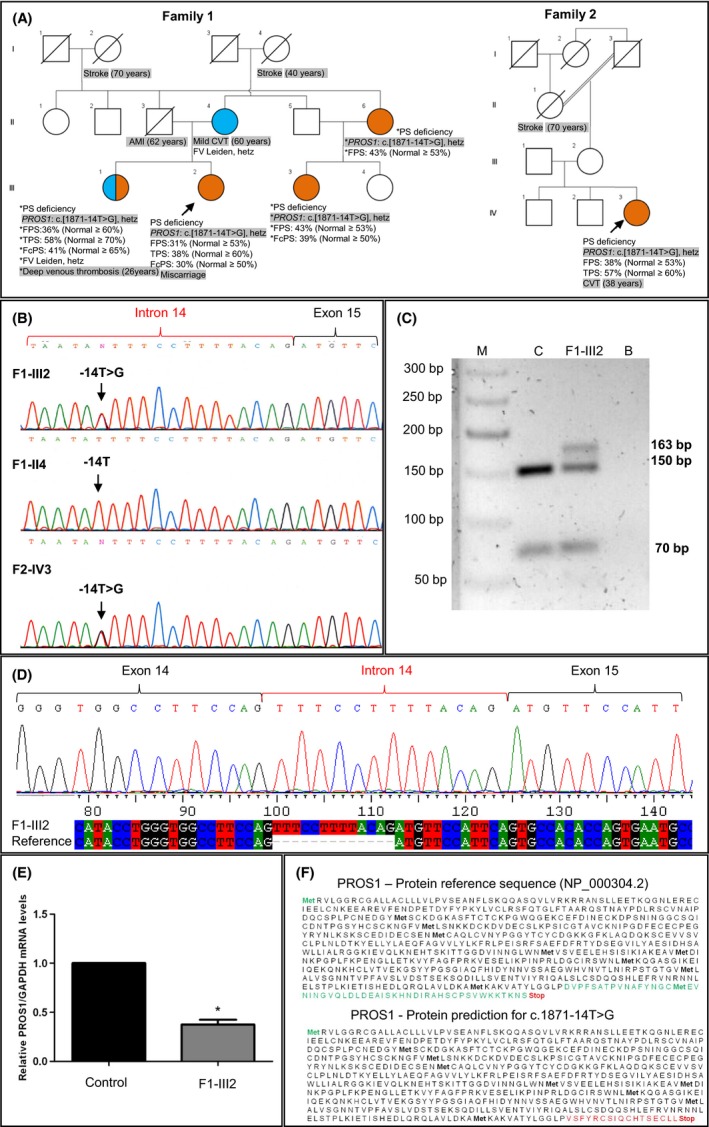
(A) Pedigrees from probands F1‐III2 and F2‐IV3. The arrow indicates each proband. Relevant results are below each family member: FPS, TPS, FcPS—free, total, and functional protein S, respectively. CVT, cerebral venous thrombosis; AMI, acute myocardial infarction. *Clinical diagnosis and laboratory analysis performed elsewhere. Orange and blue—stand for PS deficiency and FV Leiden heterozygosity, respectively. Heterozygosity (hetz) for *PROS1* variant c.1871‐14T>G and the clinical condition are shadowed in gray. (B) DNA sequence results of *PROS1* intron 14–exon 15 junctions of F1‐III2, F1‐II4, and F2‐IV3. Molecular analysis revealed that F1‐III2 and F2‐IV3 are heterozygous for *PROS1*: c.1871‐14T>G variant (NCBI sequence reference: NM_000313.3) that is predicted to activate new intronic cryptic acceptor splice site. (C) *PROS1* transcript amplification analysis of exon 14–exon 15 junctions in a 2% agarose gel. The 150‐bp fragment in the control (C) and F1‐III2 results from the amplification of the wild‐type (wt) allele, and the 163‐bp fragment found in F1‐III2 is from amplification of the mutant allele. The 70‐bp fragment in the C and F1‐III2 corresponds to *GAPDH* (internal control) amplification. M: NZYDNA ladder VI; B: Mock. (D) Chromatogram of the 163‐bp RT‐PCR fragment from patient F1‐III2. The sequence analysis revealed that c.1871‐14T>G variant resulted in the retention of the last 13 nucleotides from intron 14, inserted between exons 14 and 15 in *PROS1* mRNA. (E) Quantification of wt *PROS1* mRNA expression by real‐time PCR from patient F1‐III2. The expression levels of *PROS1* mRNA were normalized to the internal control *GAPDH* mRNA. The values of *PROS1* mRNA amplicons from F1‐III2 were compared to the control sample, arbitrarily set to 1. Patient F1‐III2 produces 40% of total wt *PROS1* mRNA when compared to a healthy control (*P* = 0.035). (F) In silico prediction of c.1871‐14G>T variant in the PROS1 protein sequence. The insertion in the mutant allele alters the reading frame and predicting a truncated protein that loses all amino acids encoded by exon 15 and gains a premature stop codon.

Two unrelated Portuguese women were referred for genetic analysis of the *PROS1* gene due to PSD and clinical conditions associated with thromboembolism (Fig. 1A, probands F1‐III2 and F2‐IV3). Plasma levels of total (TPS) and free protein S (FPS) antigen were below the lower limit of reference, suggesting type I PSD. After obtained informed consent, peripheral blood (PB) samples were collected for *PROS1* molecular analysis of patients F1‐III2 and F2‐IV3. PB was also available from patient F1‐III2 for mRNA studies, and from her mother and sister for *PROS1*, c.20210G>A Prothrombin and Factor V Leiden genetic testing.

As there were no other factors that could potentially result in a decrease in PS levels in patients F1‐III2 and F2‐IV3, hereditary type I PSD was suspected (Fig. 1A). Standard molecular analysis of *PROS1* 4 culminated with the identification of a T>G transversion at position‐14 of the acceptor splice site of intron 14 in patients F1‐III2 and F2‐IV3. This alteration was not identified in the mother of F1‐III2 (Fig. 1B), suggesting that it is of paternal origin. To our knowledge, the *PROS1* c.1871‐14T>G variant was not described associated with PSD and it is very rare in the general population (rs754929347; MAF C = 0.000009/1). Analysis of this variant using the Alamut‐v2.2 interactive biosoftware predicted the creation of a new cryptic acceptor splice site (scores: SSF = 71.78; MaxEnt = 2.32 and HSF = 76.10).

As c.1871‐14T>G has a potential effect on intron 14–exon 15 acceptor splice site, we decided to study its *PROS1* mRNA. For this purpose, RNA was isolated from fresh PB leukocytes (obtained from patient F1‐III2) and cDNA was amplified. As a result, two PCR products of 150‐bp and 163‐bp in the patient cDNA were amplified, unlike the healthy control sample in which only the wild‐type (wt) allele of 150‐bp was obtained, as expected (Fig. 1C). To better characterize the 150‐bp and 163‐bp fragments, both were separately cloned and sequenced. We found, as expected, that the 150‐bp fragment from the patient and the control sample corresponds to the wt allele with the correct junction of exons 14 and 15 (data not shown). However, the 163‐bp fragment retained the last 13 nucleotides (nt) of intron 14 (Fig. 1D). In order to quantitatively evaluate the expression level of wt *PROS1* mRNA in proband F1‐III2, we performed real‐time PCR using specific primers for the wt allele (Fig. 1E). Our results demonstrated that the patient F1‐III2 sample has only 40% of total wt *PROS1* mRNA when compared to the healthy control (*P* = 0.035).

Finally, we decided to investigate how the retention of these 13 nt in the mRNA of *PROS1* would affect the PROS1 protein. In silico analysis using the ExPASy translate tool revealed that the additional 13 nt of the mutant allele, alters the reading frame, predicting a truncated protein that loses all amino acids encoded by exon 15 (green sequence) and creates a premature termination codon (PTC) 17 amino acids downstream (red sequence) (Fig. 1F). However, nonsense‐mediated mRNA decay (NMD) may recognize and selectively degrades this mRNA carrying PTC, preventing the production of a truncated protein.

To date, only few studies have experimentally demonstrated the functional consequences of *PROS1* variants associated with type I PSD. Variants in *PROS1* leading to deleterious alleles, such as frameshift or splice‐site variants, result in significant sequence alterations and often introduce PTCs, as was demonstrated with this study. In addition, in silico splice‐site prediction tools are not enough to take definitive conclusions about the variants functional consequences. Additional mRNA‐based studies should be performed when putative splicing variants are identified. To elucidate the genetic causes underlying PSD and the heterogeneity observed in its phenotypic manifestation, it is essential to investigate the consequences of naturally occurring *PROS1* variants. Our results shed some light on the mechanism by which splice‐site variants can alter plasma PS levels.

Concerning the risk of VTE, both patients have PS levels below the lower limit of the reference range. This splicing variant probably results in a reduced synthesis of PS reflected in lower levels of FPS and TPS identified in the affected members of these two families, and possibly they have a putative increased risk of VTE as was reported in previous studies 2, 5. We also emphasize the occurrence of a thrombotic episode at an early age (26 years) in patient F1‐III1, which probably is related to the cumulative effect of the cosegregation of PROS1: c.1871‐14T>G and FV Leiden, another well‐known risk factor for thrombosis, demonstrating that the risk of thrombosis is higher in carriers of double defects.

In conclusion, we describe a novel c.1871‐14T>G splice‐site variant probably causative of type I PSD and supports the importance of the molecular and functional characterization of naturally occurring *PROS1* variants, such as mRNA‐based studies in the cases of putative splice‐site variants.

## Authorship

JM: designed and performed the mRNA experiments, analyzed the results, and wrote the manuscript. CV: performed the molecular screening of *PROS1* gene and revised the manuscript. JMC and EP: established the clinical diagnosis of the index cases of family 1 and 2, respectively. LR: analyzed the results and revised the manuscript. JG: directed the molecular screening of *PROS1*, supervised the project, analyzed the results, and revised the manuscript.

## Conflict of Interests

The authors state that they have no conflict of interests.
